# Microvesicles and Microvesicle-Associated microRNAs Reflect Glioblastoma Regression: Microvesicle-Associated miR-625-5p Has Biomarker Potential

**DOI:** 10.3390/ijms23158398

**Published:** 2022-07-29

**Authors:** Natalia Simionescu, Miruna Nemecz, Anca-Roxana Petrovici, Ioan Sebastian Nechifor, Razvan-Cristian Buga, Marius Gabriel Dabija, Lucian Eva, Adriana Georgescu

**Affiliations:** 1 Centre of Advanced Research in Bionanoconjugates and Biopolymers, “Petru Poni” Institute of Macromolecular Chemistry, 41A Grigore Ghica Voda Alley, 700487 Iasi, Romania; natalia.simionescu@icmpp.ro (N.S.); petrovici.anca@icmpp.ro (A.-R.P.); 2 “Prof. Dr. Nicolae Oblu” Emergency Clinical Hospital, 2 Ateneului Street, 700309 Iasi, Romania; nsebastian04@gmail.com (I.S.N.); bugarazvan@yahoo.com (R.-C.B.); mariusdabija.md@gmail.com (M.G.D.); elucian73@yahoo.com (L.E.); 3 Department of Pathophysiology and Pharmacology, Institute of Cellular Biology and Pathology “Nicolae Simionescu” of the Romanian Academy, 8 B.P. Hasdeu Street, 050568 Bucharest, Romania; miruna.nemecz@icbp.ro

**Keywords:** microvesicles, microRNA, glioblastoma, biomarkers, recurrence, target gene prediction, survival analysis

## Abstract

Glioblastoma (GB) is the most aggressive and recurrent form of brain cancer in adults. We hypothesized that the identification of biomarkers such as certain microRNAs (miRNAs) and the circulating microvesicles (MVs) that transport them could be key to establishing GB progression, recurrence and therapeutic response. For this purpose, circulating MVs were isolated from the plasma of GB patients (before and after surgery) and of healthy subjects and characterized by flow cytometry. OpenArray profiling and the individual quantification of selected miRNAs in plasma and MVs was performed, followed by target genes’ prediction and in silico survival analysis. It was found that MVs’ parameters (number, EGFRvIII and EpCAM) decreased after the surgical resection of GB tumors, but the inter-patient variability was high. The expression of miR-106b-5p, miR-486-3p, miR-766-3p and miR-30d-5p in GB patients’ MVs was restored to control-like levels after surgery: miR-106b-5p, miR-486-3p and miR-766-3p were upregulated, while miR-30d-5p levels were downregulated after surgical resection. MiR-625-5p was only identified in MVs isolated from GB patients before surgery and was not detected in plasma. Target prediction and pathway analysis showed that the selected miRNAs regulate genes involved in cancer pathways, including glioma. In conclusion, miR-625-5p shows potential as a biomarker for GB regression or recurrence, but further in-depth studies are needed.

## 1. Introduction

The clinical hallmarks of glioblastoma (GB), the most aggressive form of adult brain cancer, are high tumor heterogeneity, extensive vascularization, fast growth and invasiveness, poor survival and minimal response to therapy [1,2]. The latest World Health Organization (WHO) classification of tumors of the central nervous system (CNS) [3] relies on clinical presentation and histological data, as well as molecular markers for the diagnosis of GB. Therefore, GB (GB, isocitrate dehydrogenase (IDH)-wildtype) is now classified as an adult-type diffuse and astrocytic glioma, characterized by “microvascular proliferation or necrosis or telomerase reverse transcriptase (TERT) promoter mutation or epidermal growth factor receptor (EGFR) gene amplification or +7/−10 chromosome copy number changes” [3].

Traditionally, GB prognosis projections were conducted based on clinical presentation, histological features and percent of surgical resection, but recently, molecular biomarkers have been used for tumor grading and prognosis estimation [3]. Moreover, the late diagnosis of recurrence is one of the major drawbacks of GB, despite therapeutic advances and improved imaging techniques, and long-term disease management is difficult and expensive. Therefore, cost-effective, easy-to-measure and minimally invasive biomarkers for GB therapeutic response monitoring and recurrence are needed in order to better manage the disease progression.

Some of the most promising biomarkers are microRNAs (miRNAs), small non-coding RNAs which regulate gene expression post-transcriptionally through the inhibition of translation and/or the degradation of messenger RNA (mRNA) [4]. MiRNA levels are modified during disease progression [5,6] and they have been found in several body fluids, including blood, circulating in extracellular vesicles (EVs) or associated with lipoproteins or protein complexes [7]. Furthermore, miRNAs’ circulating profiles often reflect their modified expression in the tissue of origin or indicate increased intercellular communication [7,8]. MiRNAs’ quantification in biological fluids is relatively easy and cost-effective, supporting their use as biomarkers for prognosis and therapeutic response monitoring. However, to date, serum miRNA biomarkers are not applied in clinical settings for GB. 

Another promising biomarker category is represented by EVs, a heterogenous group of cell-derived lipid vesicles which exhibit specific markers of their cells of origin and transport tissue-specific cargo, including miRNAs [9,10,11,12,13,14]. EVs are intercellular communication mediators in physiological and pathological conditions [11,15], capable of crossing the blood–brain barrier [16,17]. EVs have been isolated from biological fluids [18,19,20,21,22,23], providing insights into molecular and pathological processes, as well as accessible samples for biomarker discovery. EVs exhibited diagnostic potential in different pathologies [24,25], as well as biomarker potential in the context of treatment response and disease recurrence [26,27,28,29,30,31]. Additionally, EVs contain distinctive miRNA signatures [14], which change under pathological conditions [24,32,33] and could be applied in clinical settings as biomarkers for prognosis and therapeutic response monitoring. A particular type of EV is represented by microvesicles (MVs), which are phosphatidylserine (PS)-positive EVs (100–1000 nm) with variable shapes generated by blebbing of the plasma membrane of activated cells [11,33]. MVs transport miRNAs, exhibit markers from their cells of origin, such as EGFR, its mutant variant (EGFRvIII) or epithelial cell adhesion molecule (EpCAM) [12,34], and have been shown to have diagnostic and therapeutic potential in various pathologies, including GB [24,33,35].

Despite ongoing efforts to improve the survival of GB patients, minimal advances have been made in the early detection of GB recurrence. In this study, we tried to find out whether circulating MVs and some plasma or MV-associated miRNAs could have the potential to be biomarkers for GB patients. For this purpose, we analyzed and characterized plasma MVs by flow cytometry, and we employed OpenArray screening for MV-associated miRNAs in order to identify specifically modulated miRNAs. Next, we selected several miRNAs and determined their expression in MVs and plasma, followed by target genes’ prediction and in silico survival analysis. Our final goal was to identify MV-associated miRNAs with biomarker potential for GB regression or recurrence.

## 2. Results and Discussion

### 2.1. Quantifying Circulating Plasma MVs and GB-Specific Surface Markers

**Figure 1:**
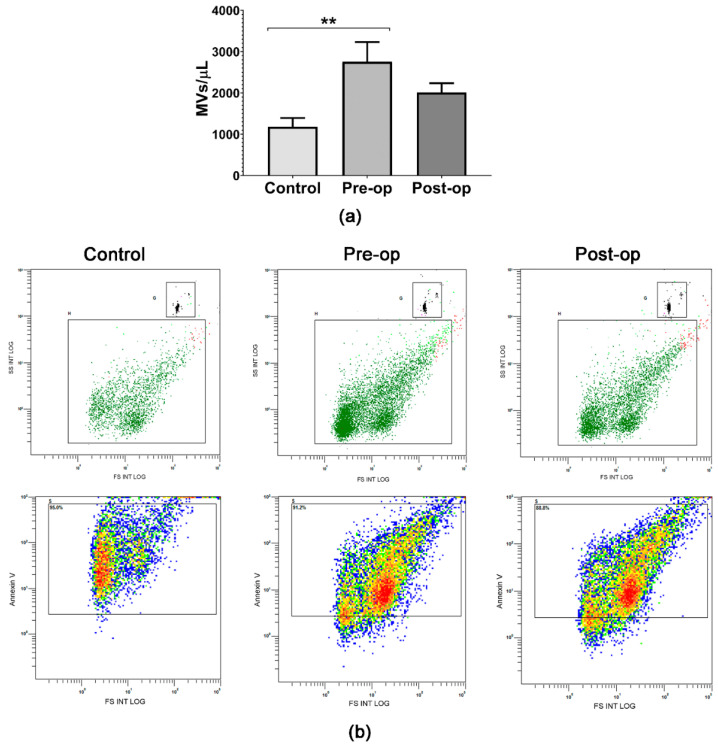
Circulating MV analysis and characterization by flow cytometry: (**a**) plasma Annexin-V-positive MV concentrations for the three groups of investigated subjects; (**b**) representative dot-plots showing the MV quantification strategy based on calibration beads 10 μm in size and 1000/µL in concentration (upper row), and purity of the isolated MV fraction based on the percentages of MVs positive for Annexin V (lower row). Data are represented as means ± standard error of the mean. ** *p* < 0.01 (One-Way ANOVA with Tukey’s multiple comparisons test).

**Figure 2:**
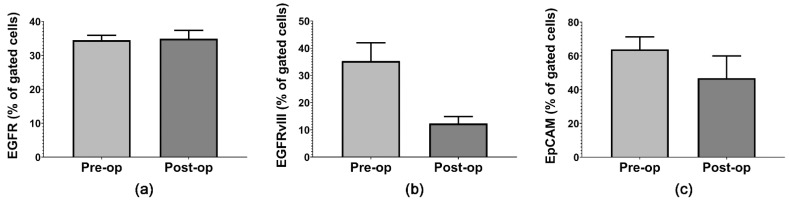
Flow cytometric analysis of GB-specific MV surface markers: (**a**) EGFR; (**b**) EGFRvIII; (**c**) EpCAM. Data are represented as means ± standard error of the mean.

Flow cytometry was used to analyze and characterize MVs isolated from plasma, including the detection of EGFR, EGFRvIII and EpCAM expression on their surface (Figure 1 and Figure 2). Control subjects had significantly lower circulating MV levels (2.34 times) compared to Pre-op GB patients (Figure 1a), in agreement with previous reports [32,36], which suggests increased intercellular communication and possibly the release of MVs from GB tumors. In addition, the circulating plasma MV concentrations (Annexin-V-positive MVs) decreased slightly (1.37 times) in GB patients’ plasma after surgical resection (Figure 1a), in agreement with Skog et al. [13]. Moreover, Koch et al. [36] showed that MVs’ number could be used to distinguish true progression from the pseudo-progression of GB. Representative dot-plots show the MV quantification and characterization strategy based on calibrated size beads and PS staining as a specific marker for MVs (Figure 1b). They also highlight the purity of MVs isolated, confirmed by PS staining with Annexin V-FITC (Figure 1b).

Our flow cytometric analysis revealed that Annexin-V-positive MVs express GB-specific markers: EGFR, EGFRvIII and EpCAM (Figure 2). There was no difference in EGFR expression on MVs’ surface between the Pre-op and Post-op groups (Figure 2a). EGFRvIII expression decreased (2.85 times) after the surgical resection of GB tumors (Figure 2b), in agreement with results reported by Skog et al. [13], and EpCAM expression decreased slightly (1.36 times) after surgery (Figure 2c). Although we did not find statistically significant differences between the Pre-op and Post-op groups, these results are consistent with previously published reports [13,34] which show high variability in surface GB markers’ expression between patients, most likely depending on the molecular profiles of their tumors.

### 2.2. miRNA Expression Profile in Circulating Plasma MVs

**Figure 3:**
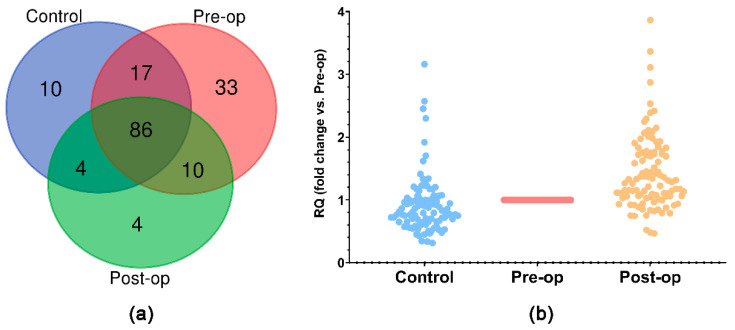
(**a**) Venn diagram of miRNAs identified in the 3 groups of subjects using TaqMan OpenArray; (**b**) scatter-plot of RQ values (fold change vs. Pre-op) for miRNAs identified in the 3 groups using TaqMan OpenArray.

The TaqMan OpenArray Human Advanced MicroRNA Panel, which allows the simultaneous quantification of 754 miRNAs, was used to determine the miRNA expression profile in circulating MVs isolated from the plasma of GB patients and Control subjects. By applying cutoffs for the good amplification quality of C_RT_ < 28 and amplification score > 1, as previously suggested [37], we identified a total of 133 miRNAs expressed in all three groups of subjects (Figure 3a). Compared to the Pre-op group, we identified 1 upregulated (fold change > 2) and 10 downregulated (fold change < 0.5) miRNAs in the Control group and 22 upregulated (fold change > 2) miRNAs in the Post-op group, presented in Figure 3b and Table 1. We found no miRNAs downregulated (fold change < 0.5) in the Post-op group compared to the Pre-op group.

**Figure 4:**
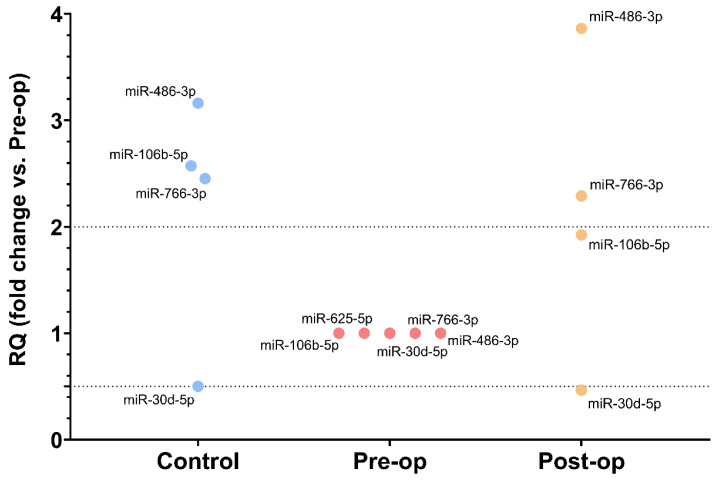
MiRNAs selected for further analysis after OpenArray profiling of MVs.

Analyzing all data obtained in the OpenArray profiling, we selected five miRNAs for further analysis (Figure 4): four miRNAs whose levels were restored to control-like levels after surgery (miR-106b-5p, miR-486-3p, miR-766-3p (upregulated) and miR-30d-5p (downregulated)) and one miRNA which was only expressed in the Pre-op group (miR-625-5p).

To date, there are few studies investigating these selected miRNAs in GB progression. It has been reported that miR-106b-5p promotes glioma cell lines’ proliferation, migration and invasion [38,39], regulates the M2 polarization of glioma-infiltrating macrophages [40] and inhibits tumor cell apoptosis in vitro and in vivo [39]. Furthermore, Liu et al. [39] showed that miR-106b-5p had significantly higher expression in GB tumor samples and cell lines than in normal brain tissue, correlated with disease grading. Regarding miR-486-3p, it was determined through in vitro and in vivo experiments that its overexpression increases chemosensitivity to temozolomide by directly targeting O6-methylguanine-DNA methyltransferase (MGMT) [41]. Furthermore, miR-486-3p was found to be overexpressed in primary glioma tissues, promoting glioma aggression, and its levels were correlated with tumor grade and poor overall survival [42]. MiR-766-3p was reported to act as a tumor suppressor in different types of cancers [43,44], but, to the best of our knowledge, its role in glioma has not been investigated. On the other hand, miR-30d-5p was demonstrated to be involved in the development and progression of different types of cancers [45], but, to the best of our knowledge, its involvement in glioma progression has not been investigated. It has been reported that miR-625-5p inhibits glioma cells’ proliferation, migration and invasion and increases their chemosensitivity [46,47]. Furthermore, Zhang et al. [46] reported significantly lower miR-625 expression in tumor samples and cell lines compared to normal brain tissue and astrocytes.

### 2.3. miRNA Quantification in Circulating MVs and Plasma

Selected miRNAs were quantified using real-time PCR in circulating plasma MVs and plasma samples from GB patients and Control subjects. The individual quantification of miR-106b-5p, miR-486-3p, miR-766-3p, miR-30d-5p and miR-625-5p in MVs confirmed the OpenArray results.

**Figure 5:**
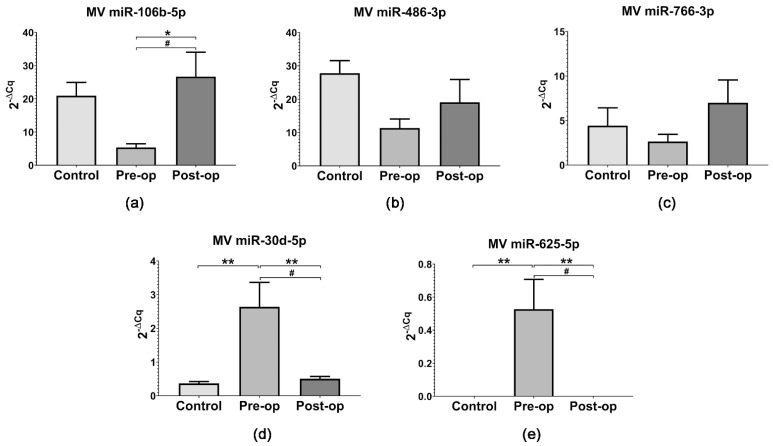
Relative expression (2^−ΔCq^) of selected miRNAs in MVs from the plasma of GB patients and Control subjects: (**a**) miR-106b-5p; (**b**) miR-486-3p; (**c**) miR-766-3p; (**d**) miR-30d-5p; (**e**) miR-625-5p. Data are represented as means ± standard error of the mean. * *p* < 0.05, ** *p* < 0.01 (One-Way ANOVA with Tukey’s multiple comparisons test), # *p* < 0.05 (paired *t*-test Pre-op vs. Post-op).

The levels of miR-106b-5p (*p* = 0.0309, paired *t*-test), miR-486-3p and miR-766-3p were upregulated in MVs from Post-op patients compared to Pre-op (Figure 5a–c), while miR-30d-5p levels (*p* = 0.0285, paired *t*-test) were downregulated after surgical resection (Figure 5d). Additionally, the levels of miR-30d-5p were significantly increased in MVs from Pre-op patients compared to Post-op (*p* = 0.0039, One-Way ANOVA) and Control MVs (*p* = 0.0013, One-Way ANOVA) (Figure 5d). In contrast to our results, Hallal et al. [48] found higher levels of miR-106b-5p and miR-486-3p and lower levels of miR-30d-5p in cavitron ultrasonic surgical aspirate (CUSA) and serum EVs from GB patients compared to Controls. However, the same study reported low levels of miR-766-3p in CUSA and serum-EVs compared to Controls and grade II-III glioma, respectively, and high levels of miR-30d-5p in CUSA EVs compared to grade II-III glioma [48], in agreement with our results. Confirming the OpenArray findings, miR-625-5p was only detected in MVs from Pre-op patients (Figure 5e). To the best of our knowledge, this is the first study which reports the quantification of miR-625-5p in circulating MVs from the plasma of GB patients. 

**Figure 6:**
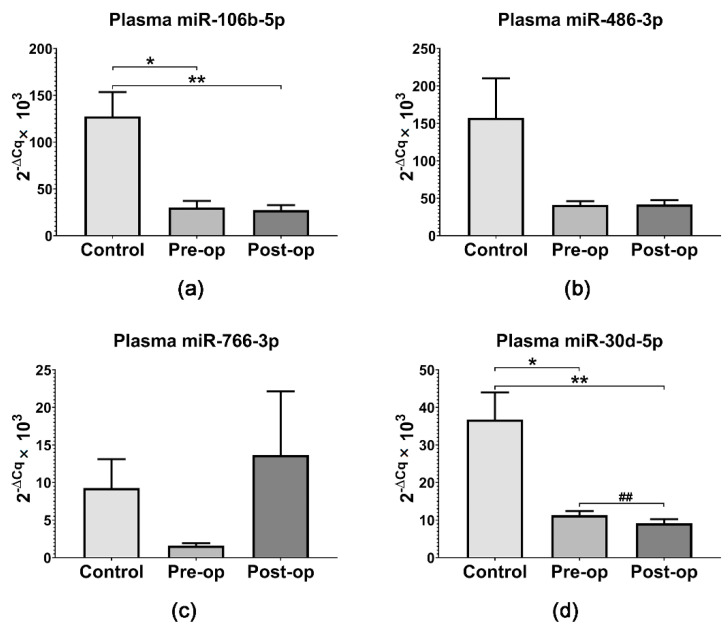
Relative expression (2^−ΔCq^ × 10^3^) of selected miRNAs in plasma from GB patients and Control subjects: (**a**) miR-106b-5p; (**b**) miR-486-3p; (**c**) miR-766-3p; (**d**) miR-30d-5p. Data are represented as means ± standard error of the mean. * *p* < 0.05, ** *p* < 0.01 (One-Way ANOVA with Tukey’s multiple comparisons test), ## *p* < 0.01 (paired *t*-test Pre-op vs. Post-op).

Interestingly, miR-106b-5p and miR-30d-5p expression was significantly lower in patients’ plasma (regardless of surgical status) compared to Control subjects’ plasma (Figure 6a,d). Additionally, plasma miR-30d-5p expression was significantly decreased (*p* = 0.0047, paired *t*-test) in GB patients’ plasma after surgery (Figure 6d). Plasma miR-486-3p expression was also decreased in patients’ plasma compared to Controls (Figure 6b). Given the fact that miR-106b-5p and miR-486-3p are significantly overexpressed in GB tumors compared to normal brain tissue [39], our results suggest a reduced export of miR-106b-5p and miR-486-3p to circulation in GB patients. The expression of miR-766-3p in plasma samples mirrored the results obtained for MVs, being decreased in Pre-op patients compared to Controls and Post-op patients (Figure 6c). Additionally, miR-625-5p was not detected in plasma samples from GB patients or Control subjects, suggesting it could be specifically transported by MVs.

### 2.4. Selected miRNAs’ Target Genes and Pathways

**Figure 7:**
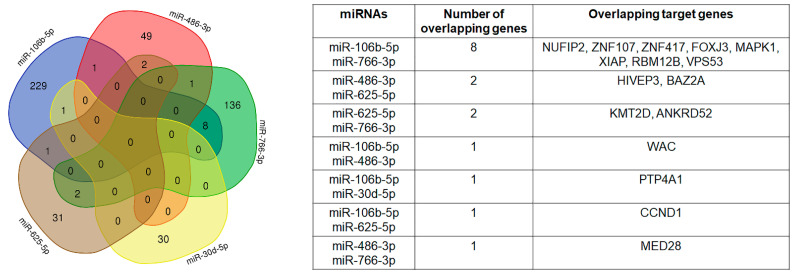
Overlapping of selected miRNA target genes.

We performed miRNA target genes’ prediction for miR-106b-5p, miR-486-3p, miR-766-3p, miR-30d-5p and miR-625-5p using the miRWalk algorithm [49]. The analysis returned the validated target genes for the five selected miRNAs: 240 for miR-106b-5p, 53 for miR-486-3p, 147 for miR-766-3p, 31 for miR-30d-5p and 36 for miR-625-5p. Overlapping of target genes occurred between a maximum of two miRNAs, as shown in Figure 7.

**Figure 8:**
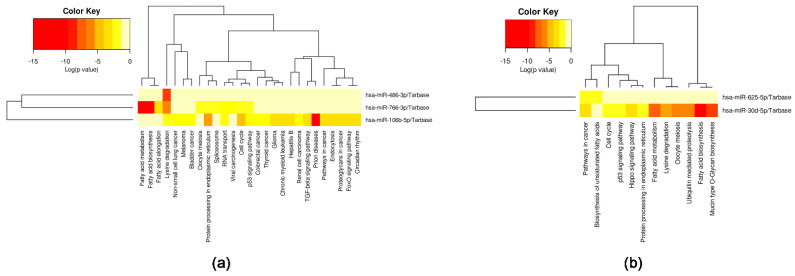
Heatmaps of significant pathways predicted by DIANA-miRPath for: (**a**) miR-106b-5p, miR-486-3p and miR-766-3p; (**b**) miR-30d-5p and miR-625-5p.

For DIANA-miRPath (v3.0) analysis [50], we grouped the selected miRNAs into two categories: miRNAs with decreased expression in Pre-op MVs (miR-106b-5p, miR-486-3p and miR-766-3p) and miRNAs with increased expression in Pre-op MVs (miR-30d-5p and miR-625-5p). The analysis of miR-106b-5p, miR-486-3p and miR-766-3p revealed validated target genes overlapping in the following pathways: endocytosis, pathways in cancer, non-small-cell lung cancer, lysine degradation and prolactin signaling pathway (Figure 8a). Analysis of miR-30d-5p and miR-625-5p showed validated target genes overlapping in glioma, pathways in cancer and melanoma (Figure 8b).

### 2.5. In Silico Survival Analysis

Using the OncoLnc online platform [51], we investigated whether the selected miRNAs’ expression was associated with GB patients’ survival. In the first stage, we obtained the Cox regression results for the selected miRNAs in GB patients, presented in Table 2. The high expression of miRNAs is correlated with the risk of death if the Cox coefficient has a positive value, while the opposite is indicated by a negative Cox coefficient [51].

The calculated Cox coefficients show that the high expression of miR-106b-5p correlates with the survival of GB patients. This, combined with miR-106b-5p levels increasing after surgical resection, suggests that MV-associated miR-106b-5p has biomarker potential for GB progression. On the other hand, the high expression of miR-766-3p is correlated with the risk of death, indicating an inverse relationship between tissue and circulating levels. The high expression of miR-30d-5p is correlated with the risk of death, which is in line with our results that miR-30d-5p expression is decreased in GB patients’ MVs after surgery. However, these correlations are weak and lack statistical significance (Table 2). On the other hand, the high expression of miR-625-5p correlates significantly (0.333, *p* = 0.035) with the risk of death, being in agreement with our results for miR-625-5p expression in MVs being restricted only to the Pre-op group. The OncoLnc platform did not return any results for miR-486-3p in GB.

**Figure 9:**
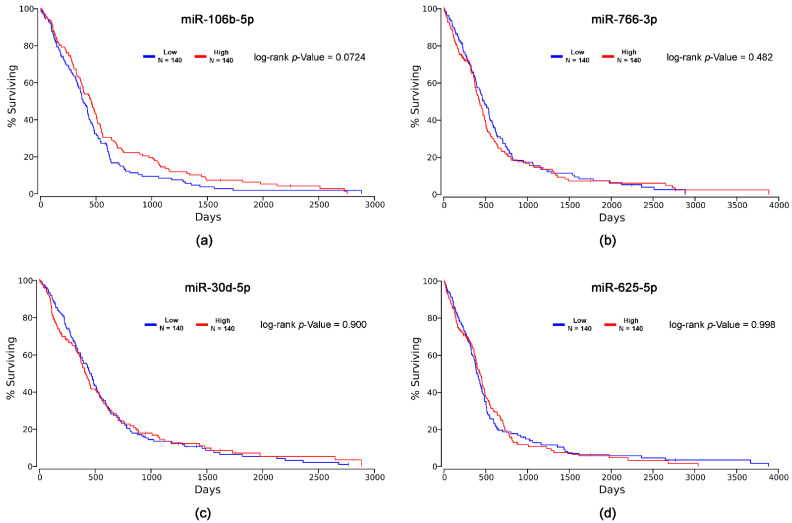
Kaplan–Meier curves and log-rank *p*-values for: (**a**) miR-106b-5p; (**b**) miR-766-3p; (**c**) miR-30d-5p; (**d**) miR-625-5p.

In the second stage, we obtained the Kaplan–Meier curves and log-rank *p*-values for each miRNA, estimating the association between miRNA expression and GB patients’ survival (Figure 9). We observed the longer survival of patients with high levels of miR-766-3p, as well as of patients with low levels of miR-625-5p. However, the log-rank *p*-values were not significant. There was no difference between the survival time of patients with low or high expression levels of miR-106b-5p and miR-30d-5p.

### 2.6. Study Limitations

There are some limitations to our study that we feel we need to mention, namely: (1) freezing the platelet-poor plasma at −80 °C and its subsequent thawing can affect the integrity of MVs obtained by successive centrifugations and characterized by flow cytometry. This problem was prevented by the easy defrosting of samples at room temperature. (2) The contamination of MVs with blood components may exist. To avoid this situation, MVs were washed two times with PBS. (3) There was a risk of incorrectly counting MVs due to contamination of the samples rich in MVs (obtained at 20,000 g) with a small number of apoptotic bodies. The use of counting beads in flow cytometry experiments allowed us to set the MV gate according to their size distribution. (4) Although MVs isolated from plasma were washed with PBS several times, it is possible some plasma miRNAs could have been carried over. However, the specificity and sensitivity of the TaqMan technology mitigates this potential limitation. (5) Survival analysis using the OncoLnc platform was performed using normalized microarray values of miRNAs from tissue samples, and these values may not always correlate with circulating levels. Further studies are needed in this regard, and bio-banks containing body fluid samples from GB patients would be very useful.

## 3. Materials and Methods

### 3.1. Study Design and Sample Collection

Patients with GB from the North East region of Romania were recruited after presenting with specific symptomatology [52] at “Prof. Dr. Nicolae Oblu” Emergency Clinical Hospital, Iasi. Patients’ selection was conducted without discrimination in terms of gender, ethnicity or religion. Exclusion criteria were non-compliant patients, autoimmune diseases or acute infections (including SARS-CoV-2). Patients underwent the typical diagnosis protocol, including anamnesis, neurological examination, routine blood tests and brain magnetic resonance imaging (MRI). Next, patients underwent the maximum safe surgical resection (aided by tumor fluorescence induced by 5-aminolevulinic acid (5-ALA)) [53]. Healthy volunteers (Controls) were recruited from the staff of the Centre of Advanced Research in Bionanoconjugates and Biopolymers, “Petru Poni” Institute of Macromolecular Chemistry, Iasi. The study was conducted according to the guidelines of the Declaration of Helsinki and approved by the Ethics Committee of “Prof. Dr. Nicolae Oblu” Emergency Clinical Hospital (no. 19092/21.11.2019). Informed consent was obtained from all subjects involved in the study.

Peripheral blood was collected on tripotassium ethylenediaminetetraacetic acid (K_3_-EDTA) anticoagulant from Control subjects (one time) and from GB patients before (Pre-op) and one week after the surgical resection of the tumor (Post-op). Peripheral blood was centrifuged at 2500× *g* for 10 min at 4 °C to collect the platelet-poor plasma (PPP) from the supernatant, which was then aliquoted and stored at −80 °C until further processing. Hemolyzed blood samples were not included in the study.

### 3.2. MV Isolation

Total circulating MVs were isolated from plasma by sequential centrifugations, using a previously published protocol [35] with some modifications. The platelet-poor plasma (PPP) was centrifuged at 16,000× *g* for 5 min at 4 °C to remove residual platelets, apoptotic bodies and collect the platelet-free plasma (PFP) in supernatant [54]. MVs were then isolated from PFP by centrifugation at 20,000× *g* for 90 min at 4 °C, and pelleted MVs were washed twice (20,000× *g*, 90 min, 4 °C) with Phosphate-Buffered Saline (PBS), re-suspended in PBS and stored at −80 °C until further analysis. For miRNA OpenArray profiling in MVs, equal amounts of plasma were pooled for each group: Control (*n* = 5), Pre-op (*n* = 5) and Post-op (*n* = 5), and MVs were isolated using the protocol described above.

### 3.3. MV Characterization by Flow Cytometry

MVs were analyzed by flow cytometry using a Gallios Flow Cytometer (Beckman Coulter Life Sciences, CA, USA). Resuspended MVs (10 µL) were mixed with 10 µL of counting beads (1000 beads/µL, 10 µm diameter) and 100 µL of PBS and counted for 60 s. The dot-plot representations (X = forward-scatter intensity, Y = sidescatter intensity) were analyzed in order to determine the number of MVs (0.1–1 µm diameter) in the samples according to the calculation formula: MVs as events/µL = [(MV count/bead count) × bead concentration/µL] × MV purity/100, as previously described [35]. In order to determine MV purity, 10 µL of resuspended MVs was incubated with 2.5 µL of Annexin V antibody (Annexin V Monoclonal Antibody (VAA-33), FITC, eBioscience, Thermo Fisher Scientific, Waltham, MA, USA) in the presence of 2 mM CaCl_2_ for 40 min at room temperature in the dark, diluted with 100 µL of PBS and analyzed by flow cytometry, as previously described [35]. The isotype control experiments were performed as well.

The expression of GB-specific surface markers on MVs was determined using antibodies against EGFR (EGFR monoclonal antibody (ICR10), PE, Invitrogen, Thermo Fisher Scientific, Waltham, MA USA), EGFRvIII (EGF Receptor vIII (D6T2Q) XP^®^ Rabbit mAb coupled with anti-rabbit IgG (H + L), F(ab’)2 Fragment (Alexa Fluor^®^ 647 Conjugate), Cell Signaling Technology, Danvers, MA, USA) and EpCAM (CD326 (EpCAM) Monoclonal Antibody (1B7), PE, eBioscience, Thermo Fisher Scientific, Waltham, MA, USA). In brief, 10 µL of resuspended MVs was incubated with 2.5 µL of Annexin V antibody and surface marker antibody, diluted at the optimal concentration (according to the manufacturer’s instructions), for 40 min at room temperature in the dark, diluted with 100 µL of PBS and analyzed with a 5000-event cutoff. Data were analyzed with Kaluza Flow Cytometry Analysis Software v2.1 (Beckman Coulter Life Sciences, Indianapolis, IN, USA).

### 3.4. RNA Isolation

Total miRNAs were isolated from 200 µL of plasma or purified MVs (resuspended in 200 µL of PBS) using the miRNeasy Serum/Plasma Advanced kit (Qiagen, Hilden, Germany), according to the manufacturer’s instructions. For technical normalization purposes, 25 fmol of synthetic cel-miR-39 (Qiagen, Hilden, Germany) was added as spike-in during miRNA isolation from plasma and MVs, as previously described [55]. Purified miRNAs were eluted with 18 μL of RNase-free water and stored at −80 °C until further analysis.

### 3.5. OpenArray Profiling of miRNAs in MVs

For miRNA OpenArray profiling, miRNAs purified from pooled plasma-derived MVs were used. A poly(A) tailing reaction, adaptor ligation reaction, reverse transcription (RT) and miR-amplification reactions were performed on a Veriti 96-Well Thermal Cycler, using the TaqMan Advanced miRNA cDNA Synthesis Kit (both from Applied Biosystems, Thermo Fisher Scientific, Waltham, MA, USA), according to the manufacturer’s protocol. MiRNA profiling was performed using the TaqMan OpenArray Human Advanced MicroRNA Panel and the TaqMan OpenArray Real-Time PCR Master Mix on a QuantStudio 12K Flex Real-Time PCR System with OpenArray block and an AccuFill System (all from Applied Biosystems, Thermo Fisher Scientific, Waltham, MA, USA), according to the manufacturer’s protocol. The obtained data were analyzed using the QuantStudio 12K Flex Software v1.2 and the ExpressionSuite Software v1.3 (both from Applied Biosystems, Thermo Fisher Scientific, Waltham, MA, USA). The expression level of each miRNA was determined relative to miR-16-5p, as recommended by the manufacturer, calculated using the 2^−ΔΔCq^ method [56] and represented as the fold change (relative quantification, RQ) of Pre-op values for each target. Raw data passed all quality control checks and were filtered by applying cutoffs of C_RT_ < 28 and amp score > 1 (measures of good amplification) [37]. Significant miRNAs were identified and selected for measurement in individual MV and plasma samples.

### 3.6. Analysis of miRNA Expression in MVs and Plasma Samples

The levels of hsa-miR-106b-5p (ID 000442), hsa-miR-30d-5p (ID 000420), hsa-miR-486-3p (ID 002093), hsa-miR-625-5p (ID 002431) and hsa-miR-766-3p (ID 001986) were measured in MVs and plasma samples using the TaqMan technology. RT was performed on a Veriti 96-Well Thermal Cycler using the TaqMan MicroRNA Reverse Transcription Kit and TaqMan miRNA assays (all from Applied Biosystems, Thermo Fisher Scientific, Waltham, MA, USA). For miRNAs isolated from MVs, the TaqMan™ PreAmp Master Mix (Applied Biosystems, Thermo Fisher Scientific, Waltham, MA, USA) was used for pre-amplification without introducing amplification bias to the sample, according to the manufacturer’s instructions. Real-time quantitative PCR was performed on a QuantStudio 12K Flex Real-Time PCR System, using TaqMan miRNA assays and the TaqMan Gene Expression Master Mix (all from Applied Biosystems, Thermo Fisher Scientific, Waltham, MA, USA), according to the manufacturer’s instructions. Obtained data were analyzed using the QuantStudio 12K Flex Software v1.2 (Applied Biosystems, Thermo Fisher Scientific, Waltham, MA, USA) with the automatic Cq setting. MiRNA levels were calculated using the 2^−ΔCq^ method [56] and multiplied by 10^3^ (for plasma). The expression level of each miRNA of interest was normalized to the exogenous spike-in cel-miR-39-5p (ID 000200) for plasma or snRNU6 (ID001973) for MVs, as previously reported [35,57]. Pre-op and Post-op miRNA levels in MVs and plasma were compared in a patient-matched manner.

### 3.7. Target Gene Prediction and Pathway Analysis

Target gene prediction was performed for miRNAs selected after OpenArray profiling using the miRWalk tool v3.0 (http://mirwalk.umm.uni-heidelberg.de/, accessed on 29 June 2022), with a score cutoff of ≥0.95 and miRTarBase filter in order to only obtain validated targets [49]. Pathway analysis was conducted with DIANA-miRPath using the miRTarBase algorithm [50].

### 3.8. In Silico Survival Analysis

The OncoLnc portal (http://www.oncolnc.org, accessed on 24 June 2022) was used to determine the associations between the selected miRNAs’ expression and the survival of GB patients [51]. This platform returns multivariate Cox regression results, as well as Kaplan–Meier plots and log-rank *p*-values for the analysis [51]. The analysis was performed on data from 563 patients from the TCGA database using the upper 25% and lower 25% slices.

### 3.9. Statistical Analysis

GraphPad Prism 8 software (GraphPad Software Inc., San Diego, CA, USA) was used for statistical analysis. Data were expressed as means ± standard error of the mean and analyzed using a paired *t*-test (Pre-op vs. Post-op) or One-Way ANOVA with Tukey’s multiple comparisons test, considering *p* < 0.05 as statistically significant.

## 4. Conclusions

Circulating plasma MV levels were positively correlated with GB severity. The surgical resection of GB tumors decreased MVs’ parameters, namely their number, as well as the expression of the EGFRvIII and EpCAM receptors, but the inter-patient variability was high. The expression of miR-106b-5p, miR-486-3p, miR-766-3p and miR-30d-5p in GB patients’ MVs was restored to control-like levels after surgery: miR-106b-5p, miR-486-3p and miR-766-3p were upregulated, while miR-30d-5p levels were downregulated after surgical resection. MiR-625-5p was only identified in MVs isolated from Pre-op GB patients and was not detected in plasma. Target prediction and pathway analysis showed that the selected miRNAs regulate genes involved in cancer pathways, including glioma.

In conclusion, miR-625-5p shows potential as a biomarker for GB regression or recurrence, but further in-depth studies are required in order to establish these miRNAs as biomarkers for GB severity and to elucidate their mechanisms of action in GB progression.

## Figures and Tables

**Table 1:** Upregulated (fold change > 2) and downregulated (fold change < 0.5) miRNAs in Control and Post-op groups compared to Pre-op group.

Control		Post-op	
miRNA	Fold Change	miRNA	Fold Change
hsa-let-7f-1-3p	0.389	hsa-let-7b-5p	2.205
hsa-miR-15b-3p	0.460	hsa-miR-10b-5p	3.211
hsa-miR-22-5p	0.489	hsa-miR-30b-5p	2.651
hsa-miR-130a-3p	0.445	hsa-miR-96-5p	2.547
hsa-miR-139-5p	0.456	hsa-miR-99a-5p	2.796
hsa-miR-143-3p	0.447	hsa-miR-106a-5p	2.175
hsa-miR-199a-3p	0.408	hsa-miR-106b-5p	2.278
hsa-miR-221-3p	0.476	hsa-miR-122-5p	2.698
hsa-miR-374a-5p	0.472	hsa-miR-125a-5p	2.584
hsa-miR-423-3p	0.269	hsa-miR-142-3p	2.032
		hsa-miR-150-5p	2.586
		hsa-miR-151a-5p	2.104
		hsa-miR-192-5p	2.161
		hsa-miR-324-5p	2.816
		hsa-miR-340-5p	2.289
		hsa-miR-342-3p	2.253
		hsa-miR-345-5p	2.650
		hsa-miR-425-3p	2.532
		hsa-miR-450a-5p	4.384
hsa-miR-486-3p	2.401	hsa-miR-486-3p	3.161
		hsa-miR-766-3p	3.511
		hsa-miR-1260a	2.365

**Table 2:** Cox regression results for selected miRNAs in GB patients.

miRNA	Cox Coefficient	*p*-Value	FDR Corrected	Rank	Median Expression	Mean Expression
**miR-106b-5p**	-0.037	0.600	0.962	325	10.85	10.81
**miR-766-3p**	0.081	0.340	0.919	192	6.55	6.7
**miR-30d-5p**	0.051	0.550	0.962	296	10.2	10.24
**miR-625-5p**	0.333	0.035	0.659	27	6.63	6.66

## Data Availability

The data presented in this study are available on request from the corresponding author.
